# Editorial: Sugars and Autophagy in Plants

**DOI:** 10.3389/fpls.2019.01190

**Published:** 2019-09-30

**Authors:** Santiago Signorelli, Céline Masclaux-Daubresse, Yuji Moriyasu, Wim Van den Ende, Diane C. Bassham

**Affiliations:** ^1^Laboratorio de Bioquímica, Departamento de Biología Vegetal, Facultad de Agronomía, Universidad de la República, Montevideo, Uruguay; ^2^Faculty of Science, The School of Molecular Sciences, The University of Western Australia, Crawley, WA, Australia; ^3^Australian Research Council Centre of Excellence in Plant Energy Biology, University of Western Australia, Crawley, WA, Australia; ^4^Institut Jean-Pierre Bourgin, INRA, AgroParisTech, CNRS, Université Paris-Saclay, Versailles, France; ^5^Graduate School of Science and Engineering, Saitama University, Saitama, Japan; ^6^Laboratory of Molecular Plant Biology, KU Leuven, Leuven, Belgium; ^7^Department of Genetics, Development and Cell Biology, Iowa State University, Ames, IA, United States

**Keywords:** ATG, SnRK1, chlorophagy, TOR, RGS1

Sugars perform an important regulatory role in plants. Part of this regulatory role is exerted by the SNF1-related kinase 1 (SnRK1), which acts as an energy sensor. The target of rapamycin (TOR) kinase is a master regulator of cell growth and metabolism and is negatively regulated by SnRK1. Through these kinases, sugars can modulate metabolism according to the energetic status of the cells.

Autophagy is an important process by which cells can recycle cellular compounds to generate energy under unfavorable growth conditions. These conditions include carbon or nitrogen deficiency or a wide range of abiotic and biotic stresses. Autophagy also plays a role during leaf senescence, contributing to nitrogen mobilization to seeds. Autophagic degradation of cellular components requires their transport to lytic vacuoles, either through double-membrane vesicles called autophagosomes (macroautophagy) or through direct invagination of the vacuolar membrane (microautophagy). The best-studied type of autophagy is macroautophagy, which requires a conserved set of autophagy-related (ATG) proteins for the formation of autophagosomes and the delivery of cargo.

This Research Topic contains articles discussing the role of sugars in plant autophagy and includes one original research paper focused on the comparison of autophagic activity in shoot and roots of the resurrection plant *Tripogon loliiformis* under desiccation conditions (Asami et al.), one mini-review about the role of sugars in controlling autophagy in plants (Janse van Rensburg et al.), one mini-review on the regulatory pathways underlying plant autophagy (Yang et al.), and two mini-reviews about the degradation of chloroplasts by autophagy (Izumi et al.; Zhuang and Jiang).

Asami et al. show that autophagy can be differentially regulated in different organs of the same plant subjected to the same conditions. In particular, they showed that unlike in shoot, autophagy is not activated in roots of *T. loliiformis* during dehydration (Asami et al.). Part of this difference was attributed to the fact that roots accumulate much more sucrose and T6P, which at high levels inhibit autophagy, *via* SWEET sugar transporters. Thus, this work demonstrates the importance of analyzing autophagy in different organs before drawing general conclusions. The mini-review from Janse van Rensburg et al. highlights the relevance of the cell wall invertases, which convert sucrose into glucose and fructose extracellularly, where the produced glucose can interact with Regulator of G-protein (RGS1) to induce autophagy by a still-uncharacterized mechanism. The authors propose that autophagy is induced by SnRK1 under sugar starvation and by RGS1 under sugar excess. Besides the control of autophagy by sugars, the authors also mention the role of autophagy in regulating sugar homeostasis (Janse van Rensburg et al.), in which the selective degradation of chloroplasts or chloroplast components by autophagy plays a major role (Izumi et al.; Zhuang and Jiang).

Multiple pathways contribute to the degradation of chloroplast components by autophagy (Izumi et al.; Zhuang and Jiang), including the formation of rubisco-containing bodies (RCBs), which allow degradation of stromal proteins; ATG8-interacting (ATI) bodies, which transport chloroplast components interacting with ATG8; the transport of small starch granule-like structures (SSGLs); and the transport of entire chloroplasts, all of them being autophagy machinery-dependent (Izumi et al.; Zhuang and Jiang). Additionally, Izumi et al. discuss the contribution of chloroplast degradation to increasing levels of free amino acids, which are used by mitochondria as respiratory substrates. Zhuang and Jiang present a table of putative ATG8-interacting motif (AIM)–containing proteins and their potential role in recognition by the autophagy machinery to target the degradation of chloroplasts.

The mini-review presented by Yang et al. assesses recent findings on the regulatory pathways underlying plant autophagy at transcriptional, post-transcriptional, and post-translational levels. They mention that some transcription factors are subjected to feedback regulation, in which autophagy is activated by these transcription factors, which are in turn degraded by autophagy (Yang et al.). They also discuss the role of the histone deacetylase 9 (HDA9) in suppressing *ATG9* gene expression and the importance of phosphorylation, ubiquitination, and lipidation of ATG proteins in the control of their activity (Yang et al.).

Over the past few years, significant progress has been made in our understanding of autophagy in plants, its regulation, and the involvement of sugars as signaling molecules in this process. It is clear that there are still many outstanding questions that need to be resolved in the future. Some of the challenges arising from this research topic are: the identification of novel regulators of plant autophagy (Yang et al.); understanding how G-protein signaling promotes autophagy (Janse van Rensburg et al.); the identification of chlorophagy receptors (Zhuang and Jiang); deciphering how chloroplasts interact with the autophagy machinery (Izumi et al.); and the identification of intermediate structures during chlorophagy (Zhuang and Jiang).

## Author Contributions

SS prepared the first draft of this editorial. DB and SS revised the editorial. SS, CMD, YM, WVdE and DCB approved it for publication.

## Funding

DB is supported by grants from the National Institutes of Health (1R01GM120316-01A1) and the National Science Foundation (MCB-1714996). WE is supported by funding from FWO Vlaanderen.

## Conflict of Interest

The authors declare that the research was conducted in the absence of any commercial or financial relationships that could be construed as a potential conflict of interest.

